# Identification of Core Senescence‐Related Genes and Characterization of Comprehensive Immune Landscape in Intervertebral Disc Degeneration

**DOI:** 10.1155/ijog/2521994

**Published:** 2025-10-22

**Authors:** Qiang Xu, Zeshuang Lian, Aoting Wang, Ding Li, Ye Wang, Yu Guo, Jialin Qin, Junfang Wang, Songyun Zhao

**Affiliations:** ^1^ Wuxi Medical Center, Nanjing Medical University, Wuxi, Jiangsu Province, China, njmu.edu.cn; ^2^ Department of Orthopedics, The Affiliated Wuxi People′s Hospital of Nanjing Medical University, Wuxi, Jiangsu Province, China, njmu.edu.cn; ^3^ Department of Orthopedics, Wuxi People′s Hospital, Wuxi, Jiangsu Province, China, wuxiph.com

**Keywords:** bioinformatics analysis, immune infiltration, intervertebral disc degeneration, machine learning algorithms, senescence, single-cell RNA sequencing

## Abstract

**Background:**

Intervertebral disc degeneration (IVDD) is a musculoskeletal degenerative disease closely associated with age and immunoreaction. However, the mechanism of senescence and immune infiltration landscape in IVDD is still unclear. Our study was aimed at investigating the pivotal senescence‐related genes (SRGs) and immune cells involved in IVDD.

**Methods:**

We downloaded expression profiles by array from the GEO database and obtained 543 human SRGs from the Human Aging Genomic Resources (HAGR). Differentially expressed gene analysis, GO, KEGG, PPI network analysis, etc., were used to identify senescence‐related differentially expressed genes (SRDEGs). We then used WGCNA and machine learning algorithms to explore hub genes and validated the reliability of the results in single‐cell RNA sequencing (scRNA‐seq) and cell models.

**Results:**

We identified a core senescence‐associated differentially expressed gene, connective tissue growth factor (CTGF). RNA sequencing revealed significantly upregulated CTGF expression in the degenerative group, a finding consistently validated by both scRNA‐seq and in vitro models. CTGF demonstrated promising diagnostic value for IVDD in risk prediction and clinical detection (AUC: 0.8361, 95% CI: 0.668–0.9953). Furthermore, we observed endothelial cells, smooth muscle cells, and erythrocytes within disc tissues, suggesting postdegenerative vascular invasion into the nucleus pulposus. Notably, immune cell infiltration, including B cells, T cells, plasma cells, and macrophages, was detected in both mildly and severely degenerated nucleus pulposus tissues, with a significantly higher abundance of immune cells in the mild degenerative group compared to severe cases.

**Conclusion:**

CTGF may play a pivotal role in IVDD, advancing our understanding of disease pathogenesis and demonstrating potential as a diagnostic and therapeutic target. Notably, reduced immune cell infiltration in severe IVDD may stem from either activation of intrinsic repair mechanisms or an immune‐exhausted state. Investigating dynamic alterations in immune cell populations during IVDD progression could elucidate the critical role of immunity in disease pathogenesis and inform novel strategies for diagnosis and therapeutic intervention.

## 1. Introduction

Lower back pain is a prevalent condition affecting a significant portion of the global population, with an estimated 637 million individuals worldwide experiencing this issue [1, 2]. On the one hand, lower back pain causes physical dysfunction and a decreased quality of life for patients [3]. On the other hand, high medical expenses pose a considerable burden on the global economy [4]. The intervertebral disc is composed of the cartilage endplate, annulus fibrosus, and gelatinous nucleus pulposus (NP) tissue. Degeneration of the L4/5 intervertebral disc has the highest incidence rate in spinal disc degeneration (up to 69.1% in males and 75.8% in females) [5], and it is the main cause of lower back pain. Risk factors for this disease include age, weight, and smoking [6]. Its pathogenesis involves mechanical stress, inflammation, cell senescence, and others [7]. By delving into the molecular mechanisms underlying disc degeneration, we can enhance our comprehension of the disease and improve diagnostic and treatment strategies.

Aging is a complex physiological process characterized by the gradual accumulation of senescent cells and associated reactions, culminating in the onset of age‐related ailments such as neurodegenerative diseases [8], atherosclerosis [9], and osteoarthritis [10, 11]. Cell senescence was originally thought to occur when cells lose their ability to proliferate and differentiate after a limited number of karyokinesis, stabilizing in a state of cell cycle arrest [12]. Currently, researchers classify cell senescence into replicative senescence (RS) caused by telomere shortening and stress‐induced premature senescence (SIPS) [13]. During the process of senescence, cells transition from a transient cell cycle arrest state to a stable cell cycle arrest state and undergo DNA damage response (DDR), which drives and enhances the expression of senescence‐associated secretory phenotype (SASP) such as cytokines, chemokines, growth factors, and extracellular matrix (ECM) proteases [14], thereby altering the tissue microenvironment and leading to disease development and declining bodily functions. Studies have shown that SASP factors like IL‐1*α*/*β*, IL‐6, and TGF‐*β* play important roles in the process of intervertebral disc degeneration (IVDD) [15]. The key senescence gene p16^INK4a^ is highly expressed in human IVDD and senescence animal models [16], indicating the significant role of senescence in IVDD. However, the exact underlying mechanisms still require further exploration.

Current evidence indicates a close interdependence between senescence and the immune microenvironment, both playing pivotal roles in IVDD [17]. Degenerated disc tissues exhibit immune activation with macrophage infiltration, generating inflammatory mediators that upregulate the expression and activity of ECM‐degrading enzymes, thereby exacerbating IVDD [18]. Senescent nucleus pulposus cells (NPCs) secrete SASP factors (e.g., IL‐1*α*/*β*, IL‐6, and TGF‐*β*), which remodel the immune microenvironment of degenerated discs and accelerate degradation. The senescence marker p16^INK4a^ demonstrates elevated expression in human degenerated discs and aging animal models [16]. Senescent NPCs secrete SASP factors (e.g., IL‐1*α*/*β*, IL‐6, and TGF‐*β*), which remodel the immune microenvironment of degenerated discs and accelerate degradation [15]. SASP components (including CXCL1 and CCL2) recruit immune cells such as macrophages, triggering chronic inflammation while suppressing tissue repair [19, 20]. Collectively, senescence and immune dysregulation form interconnected drivers of IVDD pathogenesis, though their precise mechanistic crosstalk requires further investigation.

**Figure 1:**
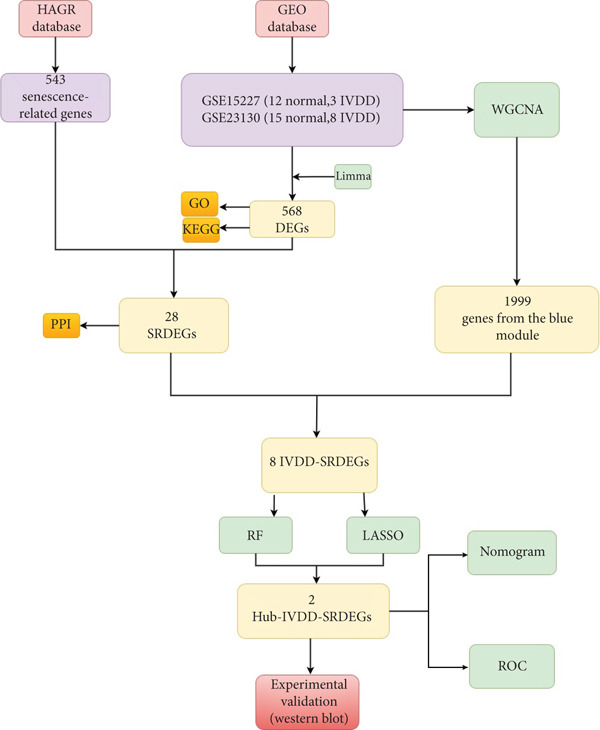
Work flowchart for this study.

In this study, we integrated two datasets and ultimately obtained gene expression data of 38 human intervertebral discs. We also downloaded senescence‐related genes (SRGs) from Human Aging Genomic Resources (HAGR). Then, we applied weighted gene coexpression network analysis (WGCNA), two kinds of machine learning, and other methods to identify senescence‐related hub genes and diagnostic biomarkers of IVDD. In the end, we validated the reliability of our results by single‐cell RNA sequencing and western blotting. The overall workflow of this study is depicted in Figure 1.

## 2. Materials and Methods

### 2.1. Gene Expression Datasets Screening and Processing

We obtained gene expression profiling by array (GSE23130 and GSE15227) of degenerated intervertebral disc tissues and normal intervertebral disc tissues from the Gene Expression Omnibus (GEO). The details of the datasets are shown in Table 1. After removing missing values, we performed background correction and normalization on the datasets using the R package limma. We then merged the two datasets and used the R package SVA to eliminate batch effects between the two datasets. Based on the Thompson grade, we divided 38 samples into the degeneration group (Thompson Grades IV and V) and the control group (Thompson Grades I–III). A two‐dimensional principal component analysis (PCA) clustering plot was used to show the sample grouping after batch effect removal. The merged and batch‐effect‐corrected gene expression data were used for subsequent analysis.

**Table 1:** Descriptive statistics.

**GEO (ID)**	**Platform**	**Tissue (*Homo sapiens*)**	**Samples (number)**	
			**Control**	**Degenerative**
GSE23130	GPL1352	Disc tissue	15	8
GSE15227	GPL1352	Disc tissue	12	3
GSE244889	GPL24676	Nucleus pulposus	4	3

### 2.2. Download and Collation of SRGs

In this study, human SRGs were acquired from the HAGR (https://genomics.senescent.info/), comprising GenAge (307 genes) and CellAge (279 genes). A total of 543 SRGs were amalgamated and deduplicated for subsequent analysis.

### 2.3. Identification of Differentially Expressed Genes (DEGs) and Functional Enrichment Analysis

DEG analysis was performed on the gene expression data using the R package limma. |logFC| > 1 and *p* *v*alue < 0.05 were selected as the criteria for screening to identify DEGs between the two groups. Heatmap and volcano plot were generated to visualize the DEGs. The R package clusterProfiler was utilized for Gene Ontology (GO) and Kyoto Encyclopedia of Genes and Genomes (KEGG) enrichment analysis of the DEGs, with a pvalueCutoff set to 0.01.

### 2.4. Identification of Senescence‐Related Differentially Expressed Genes (SRDEGs) and Protein–Protein Interaction (PPI) Network

From the list of DEGs, the expression matrix of SRGs was extracted and named SRDEGs. The SRDEGs were imported into the PPI analysis website STRING (https://cn.string-db.org/) to construct a PPI network. An interaction score > 0.7 was used as the filtering condition for selecting PPIs in the network.

### 2.5. WGCNA and Screen for Senescence‐Related Differentially Expressed Genes Highly Correlated With Intervertebral Disc Degeneration (IVDD‐SRDEGs)

The WGCNA is a method that identifies genes with highly coordinated expression patterns and forms modules to explore the relationships between gene networks and phenotypes of interest, aiming to identify hub genes. In this study, genes were ranked based on absolute median deviation from high to low, and the Top 8000 genes were selected. The “pickSoftThreshold” function was used to filter and validate the optimal soft threshold power (*β*), which was then used to transform the topological overlap matrix (TOM). Genes were grouped into different modules, and the module with the strongest correlation to IVDD and *p* value < 0.05 was selected. Genes from this module were extracted, and the intersection with SRDEGs was taken to obtain IVDD‐SRDEGs.

### 2.6. Identification of Candidate Hub Senescence‐Related Differentially Expressed Genes Highly Correlated With Intervertebral Disc Degeneration (Hub‐IVDD‐SRDEGs) via Machine Learning

Two machine learning algorithms, LASSO and random forest, were employed to screen and identify hub‐IVDD‐SRDEGs. The glmnet function from the R package glmnet was used with an alpha value of 1 to obtain the optimal lambda through 10‐fold cross‐validation. The lambda.min was used to build a LASSO regression model on the IVDD‐SRDEG expression matrix to extract the feature genes related to senescence. Random forest is an ensemble learning method that utilizes multiple decision trees to address classification and regression problems. Setting the number of decision trees to 1000, gene importance was calculated to identify senescence‐related feature genes. The feature genes selected by both machine learning methods were defined as hub‐IVDD‐SRDEGs. Visualized hub‐IVDD‐SRDEGs selected by two machine learning methods were visualized using the R package Venn.

### 2.7. Diagnostic Value of Hub‐IVDD‐SRDEGs in IVDD and Normal Samples

The nomogram was created by the R package rms to depict the occurrence rate of IVDD based on two hub genes. The accuracy of the nomogram was assessed using calibration curves, clinical decision curves, and clinical impact curves, while the ROC curve was employed to evaluate the predictive ability of the model.

### 2.8. Verifying Expression Levels of Hub Genes and Characterization of Comprehensive Immune Landscape Through Single‐Cell RNA Sequencing

In this study, we analyzed scRNA‐seq datasets (GSE144889) from NP tissues, differentiating between mildly (Grades I–II) and severely (Grades III–V) degenerative samples based on the Pfirrmann grading system. We filtered the cells with nFeature_RNA > 200, nFeature_RNA < 4000, and percent_mito < 5 to ensure data quality. The data were then standardized and normalized using the R package Seurat. We identified the Top 2000 highly variable genes for PCA. To address batch effects, we employed the harmony package, and cells were clustered using the FindClusters function (dims = 1 : 18, resolution = 0.5). Cell annotation was achieved by integrating marker genes identified through the FindAllMarkers function with classical marker genes specific to each cell type. NPCs and macrophages were subsequently isolated for further clustering analysis and subpopulation identification. Cell clusters were annotated by integrating canonical markers reported in prior literature with cluster‐specific marker genes identified via the FindAllMarkers function. Pseudotime trajectory analysis was then performed using Monocle2 to delineate NPCs′ differentiation dynamics.

### 2.9. Expression Levels of Hub Gene and Validation in Cell Models

Human NPCs were purchased from Procell (Catalog CP‐H097; Wuhan, China), while rat NPCs were obtained from Procell (Catalog CP‐R145; Wuhan, China). The cells were cultured in DMEM‐F12 medium (BL305A, Biosharp, Hefei, China) supplemented with 10% fetal bovine serum (FBS, VivaCell, Shanghai, China) and treated with IL‐1*β* (10 ng/mL, Aladdin, Shanghai, China) to establish a cell model of IVDD. The NPCs were cultured at 37°C with 5% CO_2_ in a CO_2_ incubator for 24 h. The cells were harvested at low temperature, lysed in RIPA buffer, and finally centrifuged to obtain total protein. The protein expression level was measured using a BCA protein quantification kit (B665595, Aladdin, Shanghai, China). Total protein was first separated in 10% SDS‐PAGE and then transferred to a PVDF membrane. The PVDF membrane was incubated overnight at 4°C with the following primary antibodies: *β*‐actin (Cat No. 20536‐1‐AP, Proteintech, Wuhan, China, 1:1000 dilution for WB), connective tissue growth factor (CTGF) (Cat No. 25474‐1‐AP, Proteintech, Wuhan, China, 1:5000 dilution for WB), and collagen II (ab 307674, abcam, Cambridge, 1:1000 dilution for WB). A secondary antibody, goat anti‐rabbit IgG (AP307P, Sigma‐Aldrich, United States), was used, and the membranes were incubated at room temperature for 90 min. After preparing the ECL substrate by mixing Component A and Component B at a 1:1 ratio, the protein expression was observed using an automated chemiluminescence imaging system (Tannon‐5200, China). Cellular senescence was assessed using a Senescence *β*‐Galactosidase Staining Kit to evaluate the expression of senescence‐associated *β*‐galactosidase (SA‐*β*‐gal) in both degenerative and control group cellular models.

### 2.10. Statistical Analysis

The statistical analysis was performed using R 4.3.3. Based on the normality of the data, Student′s *t*‐test or the Mann–Whitney test was used to analyze the differences between two groups of data. For categorical variables, the chi‐square test was used. A *p* value less than 0.05 was considered to indicate a statistically significant difference between different groups of data.

## 3. Results

### 3.1. Data Preprocessing

First, we downloaded expression profiling by array of intervertebral disc tissue (GSE23130 and GSE15227) from the GEO database and then merged the two datasets. The box plots in Figure 2a,b show that before removing batch effects, there was a significant difference in the sample distribution between the two datasets. After eliminating batch effects, the median distribution in samples from the two datasets tended to be similar. Next, based on the Thompson grade, we divided the samples into the degenerated group (Thompson Grades IV and V) and control group (Thompson Grades I–III). The two‐dimensional PCA clustering plot shows there are differences between the two groups after removing batch effects (Figure 2c).

Figure 2Data preprocessing. (a, b) Box plots showing sample distribution of the datasets before and after removing batch effects. (c) PCA of gene expression profiling after removing batch effects.

**Figure:**
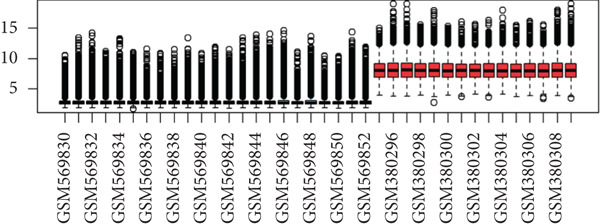
(a)

**Figure:**
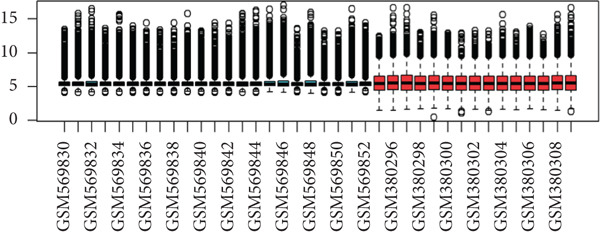
(b)

**Figure:**
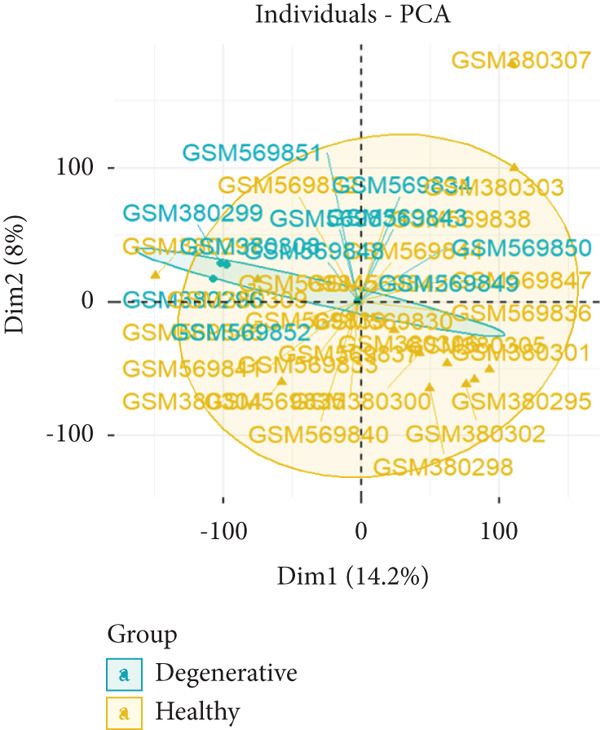
(c)

### 3.2. Identification and Functional Enrichment Analysis of DEGs

After merging and preprocessing the datasets, we conducted DEG analysis on a gene expression matrix consisting of 11 intervertebral disc tissues with Thompson Grades IV and V and 27 intervertebral disc tissues with Thompson Grades I–III. We used the R package limma with adjusted |logFC| > 1 and *p* *v*alue < 0.05 criteria, resulting in a total of 568 DEGs, including 518 upregulated genes and 50 downregulated genes, as shown in the heatmap and volcano plot (Figure 3a,b). To better understand the roles of the DEGs in IVDD, we performed GO and KEGG enrichment analyses on the 568 DEGs using clusterProfiler (Figure 3c,d). The GO enrichment analysis revealed that the DEGs were involved mainly in biological processes such as cytoplasmic translation, organonitrogen compound metabolism, biosynthetic processes, peptide biosynthetic processes, and peptide metabolic processes. They are associated with cellular components like cytosolic ribosomes, ribosomal subunits, extracellular exosomes, extracellular vesicles, and extracellular organelles. The molecular functions of the DEGs included structural constituent of ribosomes, structural molecule activity, ECM structural constituent, RNA binding, and collagen binding. The KEGG analysis revealed that the DEGs were enriched mainly in pathways related to ribosomes, coronavirus disease (COVID‐19), and amyotrophic lateral sclerosis.

Figure 3Identification and functional enrichment analysis of DEGs. (a, b) Heatmap and volcano plot showing the DEGs between the degenerated group and the control group. (c) Bar plot showing enriched Gene Ontology (GO) terms for DEGs. (d) Bar plot showing enriched Kyoto Encyclopedia of Genes and Genomes (KEGG) terms for DEGs.

**Figure:**
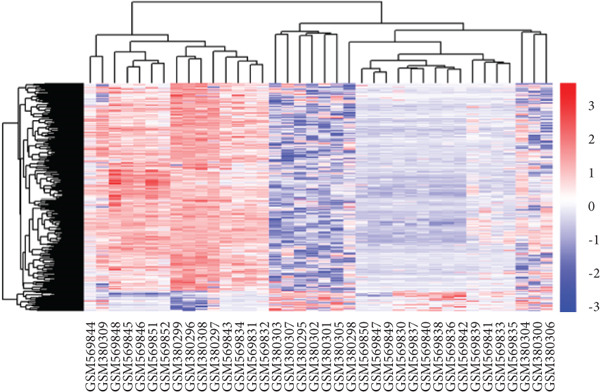
(a)

**Figure:**
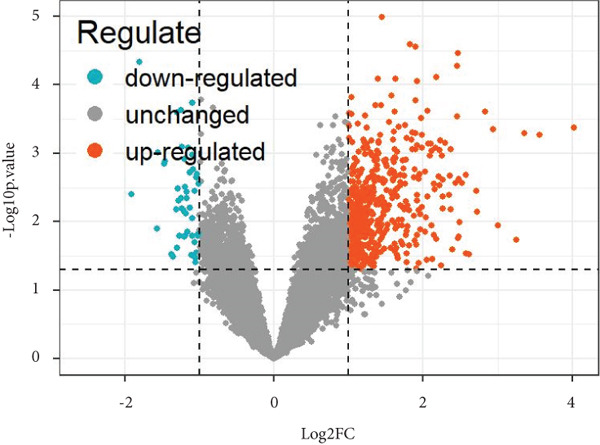
(b)

**Figure:**
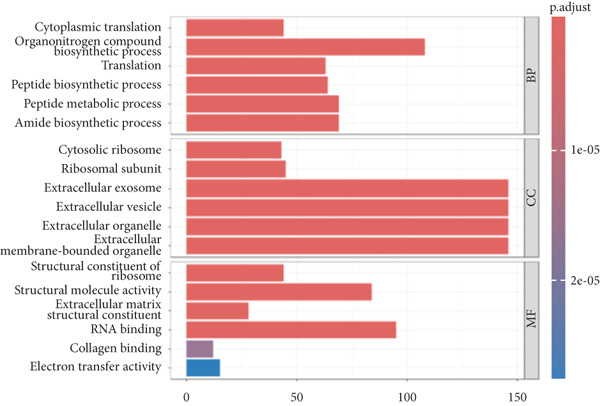
(c)

**Figure:**
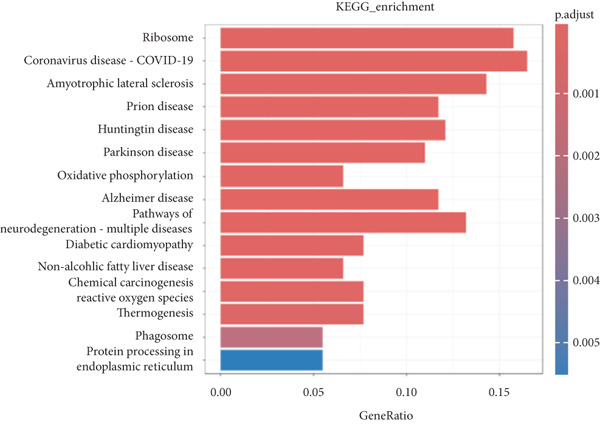
(d)

### 3.3. SRDEGs and PPI Network Analysis

DEGs and SRGs have 28 overlapping genes (Figure 4a). The PPI network analysis indicates that 28 SRDEGs closely interact at the protein level (Figure 4b).

Figure 4SRDEGs and PPI network analysis. (a) Venn diagram showing the intersection of DEGs and SRGs. (b) PPI network of the 28 SRDEGs.

**Figure:**
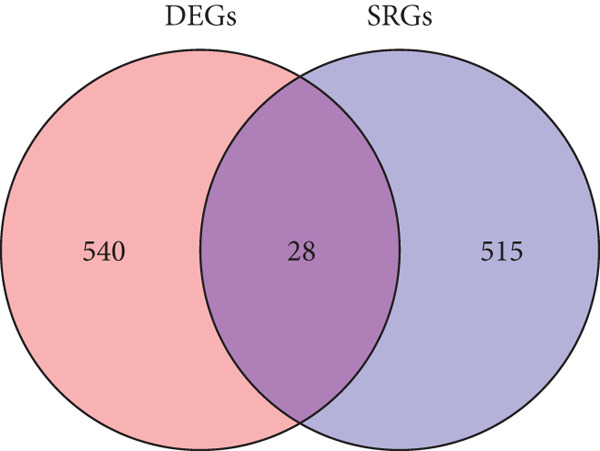
(a)

**Figure:**
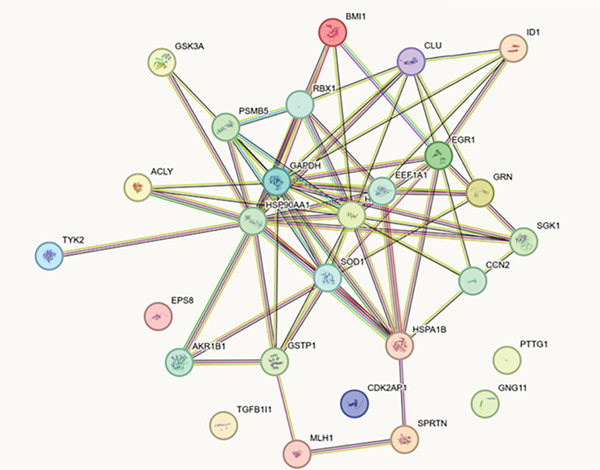
(b)

### 3.4. Identification of IVDD‐SRDEGs Using WGCNA

By utilizing the R package WGCNA, we selected the Top 8000 genes based on median absolute deviation in the expression matrix to construct a gene coexpression network. The soft threshold power (*β*) was set as 6 to build a scale‐free network, as illustrated in Figure 5a. Subsequently, cluster analysis was employed to identify highly similar modules, with a minimum module size setting of 90. This analysis resulted in the identification of five gene modules, out of which the blue module (containing 1999 genes) exhibited the highest correlation (cor = 0.43; *p* = 0.008) with IVDD, as depicted in Figure 5b,c. Genes from the blue module were extracted and overlapped with SRDEGs, leading to the identification of eight IVDD‐SRDEGs: GSK3A, CTGF, PSMB5, EEF1A1, GAPDH, HSP9OAA1, GRN, and RBX1, as shown in Figure 5d.

Figure 5WGCNA. (a) Determination of the optimal soft threshold power using the powerEstimate function resulting in a value of 6. (b) Gene clustering dendrogram. (c) Module–trait relationship heatmap. (d) Venn diagram illustrating the intersection of genes from the blue module and SRDEGs.

**Figure:**
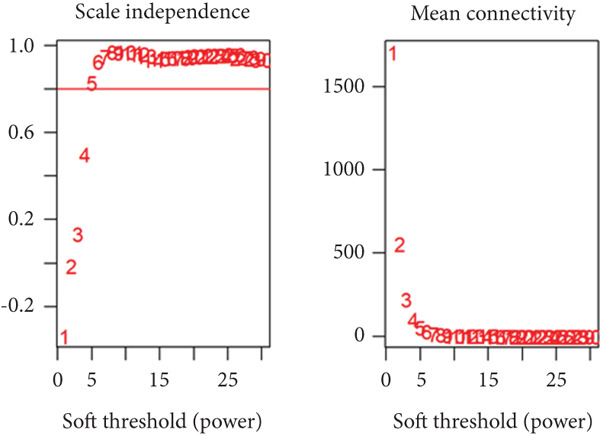
(a)

**Figure:**
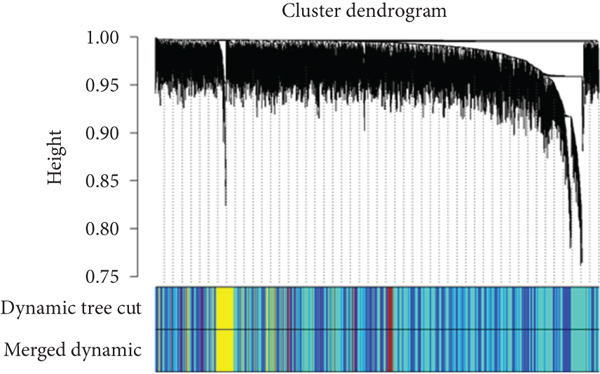
(b)

**Figure:**
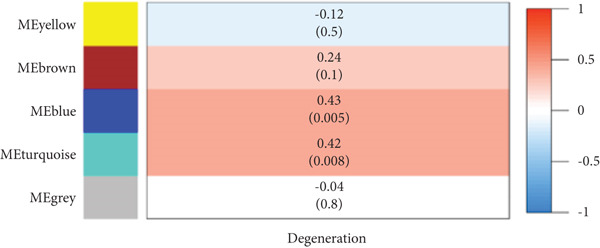
(c)

**Figure:**
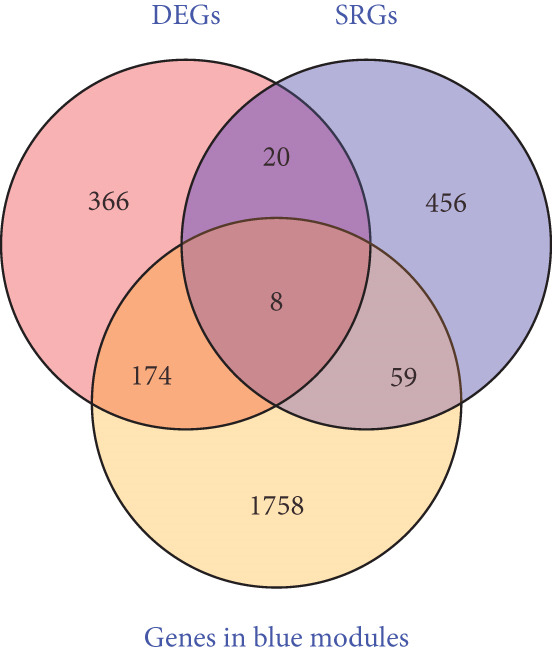
(d)

### 3.5. Identification of Hub‐IVDD‐SRDEGs Using Machine Learning

To further identify hub‐IVDD‐SRDEGs, we employed machine learning algorithms (LASSO and random forest). The LASSO identified three optional genes (CTGF, GSK3A, and GRN) from the eight IVDD‐SRDEGs (depicted in Figure 6a,b), while RF ranked the importance of the eight IVDD‐SRDEGs (shown in Figure 6c,d). Two genes overlapped between the three genes from LASSO and the Top 3 genes according to importance from RF (illustrated in Figure 6e). Ultimately, we identified two hub‐IVDD‐SRDEGs: CTGF and GSK3A.

Figure 6Machine learning for identifying hub‐IVDD‐SRDEGs. (a, b) The LASSO coefficient profiles and 10‐fold cross‐validation for optimum tuning parameter. (c, d) Random forest ranking gene importance. (e) Venn diagram showing the overlapping genes from LASSO and RF results.

**Figure:**
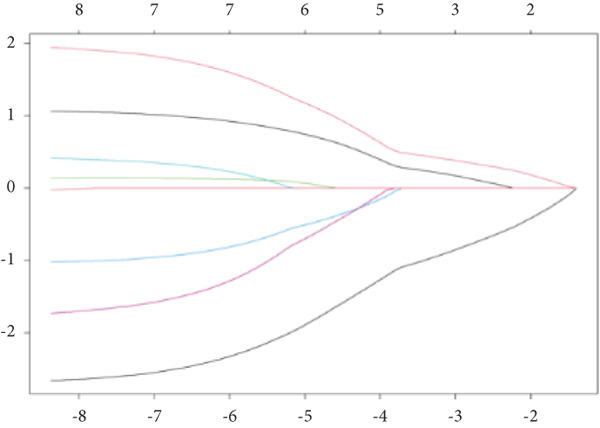
(a)

**Figure:**
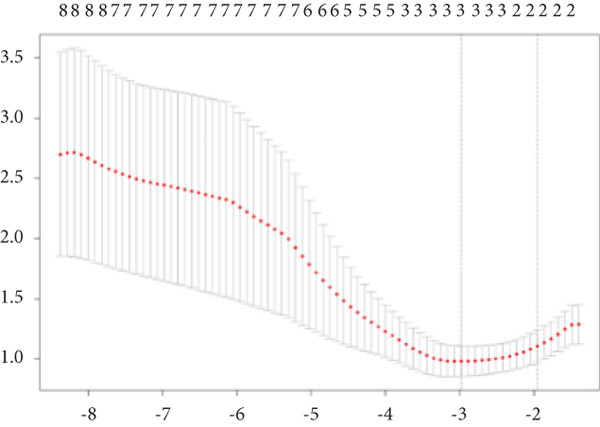
(b)

**Figure:**
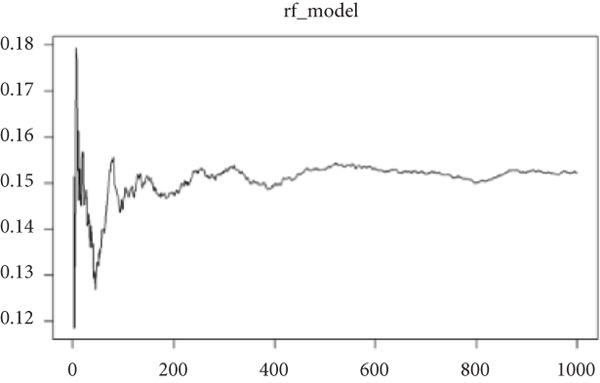
(c)

**Figure:**
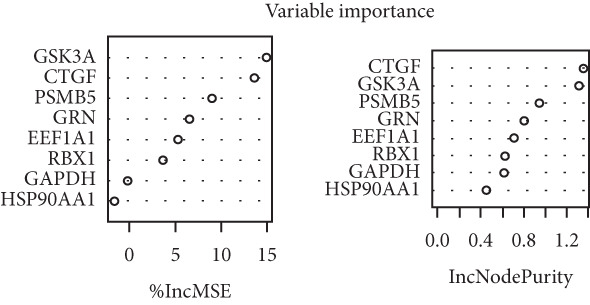
(d)

**Figure:**
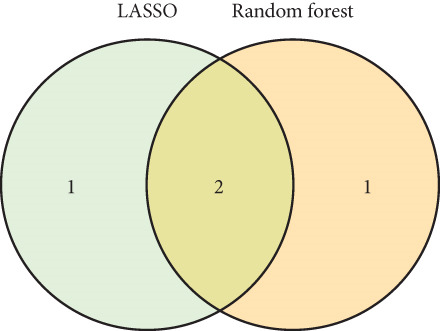
(e)

### 3.6. The Diagnostic Value of Hub‐IVDD‐SRDEGs in IVDD

We constructed a risk prediction model for IVDD using the two selected genes most strongly correlated with senescence (CTGF and GSK3A) and plotted a nomogram to demonstrate the diagnostic value of the two genes, with scores for predicting the disease risk of IVDD shown in Figure 7a. The calibration curve in the graph indicates that the model′s predictions align closely with actual occurrences when the predicted disease likelihood exceeds 60% (Figure 7b). Both the clinical decision curve (Figure 7c) and clinical impact curve (Figure 7d) clearly show the model′s accuracy in predicting disease likelihood exceeding 60%. Additionally, the ROC curves illustrate that the two genes have good diagnostic value for the disease (Figure 7e).

Figure 7Diagnostic value of hub‐IVDD‐SRDEGs in IVDD. (a) Nomogram of hub‐IVDD‐SRDEGs in the diagnosis of IVDD patients. (b) Clinical calibration curve estimating the predictive accuracy of the nomogram (the closer to the ideal dashed line, the more reliable the results are). (c) Clinical decision curve assessing the accuracy of the model (the farther the red line endpoint is from the gray line, the greater the accuracy). (d) Clinical impact curve (red solid line represents the predicted number of affected individuals at different disease probabilities; blue dashed line indicates the actual number of affected individuals at different disease probabilities). (e) ROC curve for the two hub‐IVDD‐SRDEGs.

**Figure:**
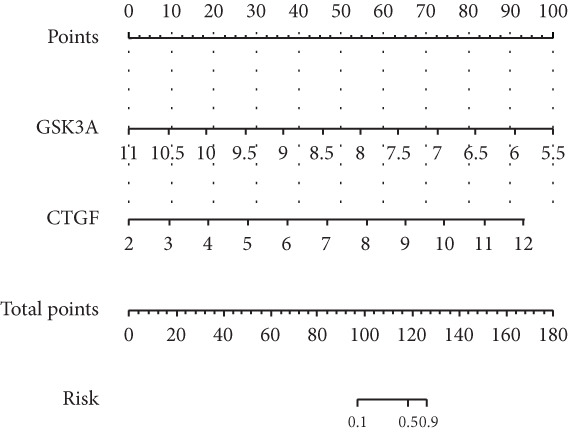
(a)

**Figure:**
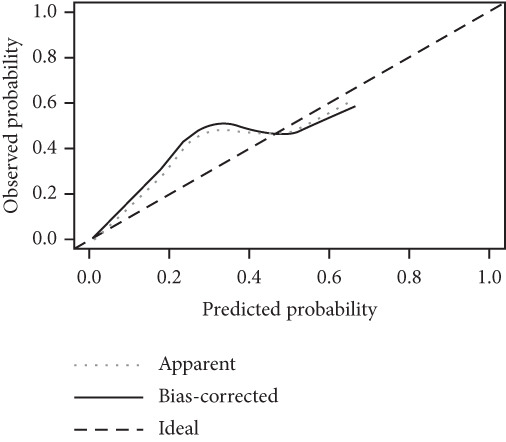
(b)

**Figure:**
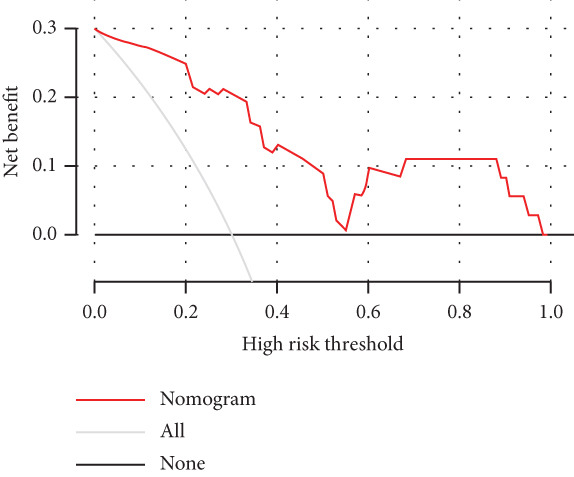
(c)

**Figure:**
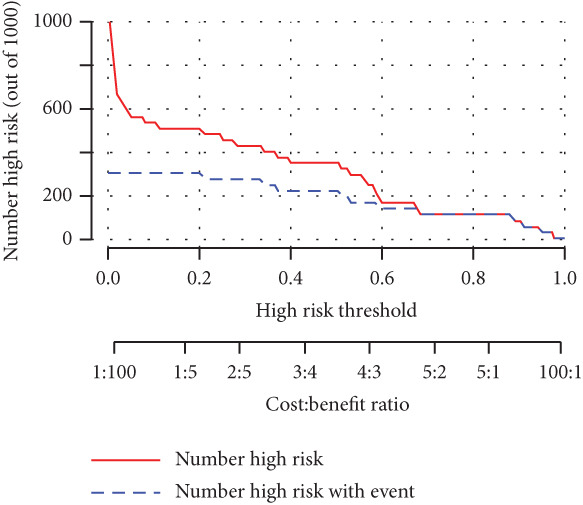
(d)

**Figure:**
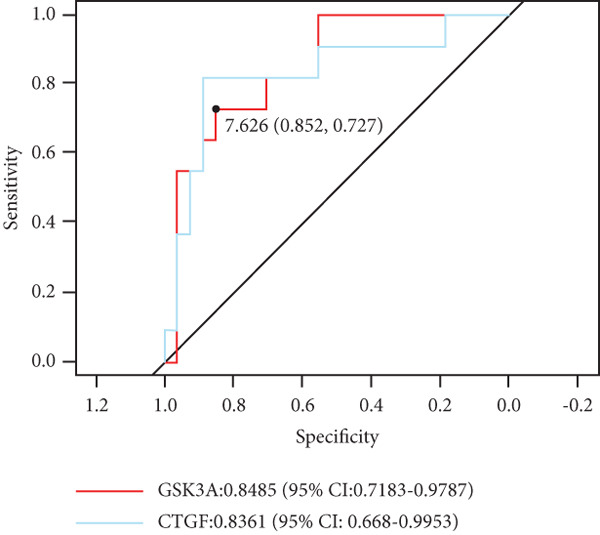
(e)

### 3.7. scRNA‐Seq Verified the Expression Levels of Hub Genes and Revealed Immune Cell Infiltration in IVDD

Utilizing unsupervised graph‐based clustering of the integrated dataset comprising 54,373 cells from seven samples, we identified 16 distinct clusters based on nearest neighbor approximations. We employed uniform manifold approximation and projection (UMAP) to visualize these clusters (Figure 8a). Cell annotation was performed by integrating both classical cell marker genes reported in the literature and those identified via the FindAllMarkers function. The resulting population partitions included NPCs (characterized by ACAN+, COL2A1+, and SOX9+), erythrocytes, endothelial cells, smooth muscle cells (SMCs), plasmablasts, T cells, B cells, neutrophils, and macrophages (Figure 8b). UMAP visualizations for the NPC marker genes (ACAN, COL2A1, and SOX9) are presented in Figures 8d, 8e, and 8f. To further elucidate the differences in cellular composition, we utilized a balloon plot to display the specific numbers of various cell types across the control and degeneration groups (Figure 8c). Notably, the counts of epithelial cells and SMCs were significantly elevated in the severely degenerative group, while the number of erythrocytes increased in the mildly degenerative group. Additionally, the infiltration of immune cells, including B cells, T cells, neutrophils, macrophages, and plasmablasts, was more pronounced in the mildly degenerative group. In terms of gene expression, GSK3A levels in NPCs were found to be low, and no significant correlation with COL2A1 expression was identified in the coexpression analysis (Figure 8g). Expression levels of GSK3A across all cell types were generally low, with no statistically significant differences observed between the two groups among NPCs. While there were variations in expression levels of B cells and SMCs between the degeneration and control groups, these levels remained extremely low (Figure 8h). Moreover, CTGF (CCN2) expression was significantly elevated in degenerative NPCs exhibiting low COL2A1 expression (Figure 8i). The box plot analysis further demonstrated a significant increase in CTGF (CCN2) expression specifically in both NPCs and endothelial cells within the degeneration group compared to control (Figure 8j). NPCs were extracted and clustered into six distinct cell clusters (Figure 8k). These clusters were annotated as effective NPCs, fibrotic NPCs, inflammatory NPCs, metabolic NPCs, and regulating NPCs based on canonical marker proteins (CHRDL2, FBLN1, IL11, DKK1, and CHI3L1) (Figure 8l,m). Pseudotime trajectory analysis using Monocle2 revealed that effective NPCs were located at the pseudotime origin, while fibrotic NPCs occupied the terminal pseudotime state (Figure 8n). Notably, degenerative disc tissues exhibited a higher proportion of fibrotic NPCs compared to controls. Both groups showed significant downregulation of ECM components (ACAN and COL2A1) at the terminal differentiation stage, with a more pronounced reduction in the degenerative group. Intriguingly, SA‐*β*‐gal (GLB1) expression displayed a biphasic pattern during differentiation and senescence—initial decline followed by upregulation. Furthermore, CCN2 expression was markedly elevated in the degenerative group compared to controls, confirming its progressive upregulation during NPC degeneration and senescence (Figure 8o). This observation directly corroborates the central hypothesis. Following macrophage subset isolation and clustering (Figure 8p), we annotated subpopulations using canonical markers: M1 (CD86) and M2 (CD163) (Figure 8q,r). Quantitative analysis revealed M1 dominance in mild degeneration and M2 predominance in severe degeneration (Figure 8s). This phenotypic shift implies distinct pathological roles: M1 macrophages drive proinflammatory cascades to exacerbate early degeneration, while M2 polarization promotes reparative responses in advanced stages—providing critical mechanistic insights for therapeutic development.

Figure 8scRNA‐seq analysis of the nucleus pulposus. (a) Visualization of clustering by UMAP plot. (b) UMAP map of the following nine cell types: NPCs, erythrocyte, endothelial, smooth muscle cells (SMCs), plasmablast, T cell, B cell, neutrophil, and macrophage. (c) Balloon plot of various cell types across the control and degeneration groups. (d–f) UMAP image of the NPC marker genes: COL2A1, SOX9, and ACAN. (g) UMAP image of COL2A1, GSK3A, and coexpression. (h) Box plot of GSK3A expression levels in all cells.  ^∗^ *p* < 0.05; ns, not significant (*p* ≥ 0.05). (i) UMAP image of COL2A1, CTGF, and coexpression. (j) Box plot of CTGF expression levels in all cells. ^∗^ *p* < 0.05 and  ^∗∗∗^ *p* < 0.05 < 0.0001; ns, not significant (*p* ≥ 0.05). (k) Visualization of NPC clusterings by UMAP plot. (l) UMAP map of NPC cell types. (m) The differentially expressed genes in each of the six NPC subclusters. (n) Monocle pseudotime trajectory showing the progression of NPCs. (o) ACAN, COL2A1, CCN2, and GLB1 expression in normal and degenerative tissues by pseudotime. (p) Visualization of macrophage clusterings by UMAP plot. (q) The differentially expressed genes M1 (CD86) and M2 (CD163). (r) UMAP map of macrophage cell types. (s) Stacked bar chart of macrophages across the control and degeneration groups.

**Figure:**
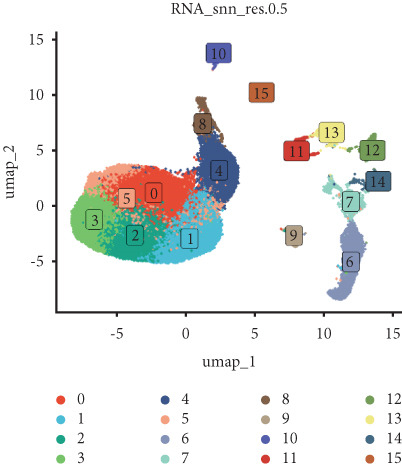
(a)

**Figure:**
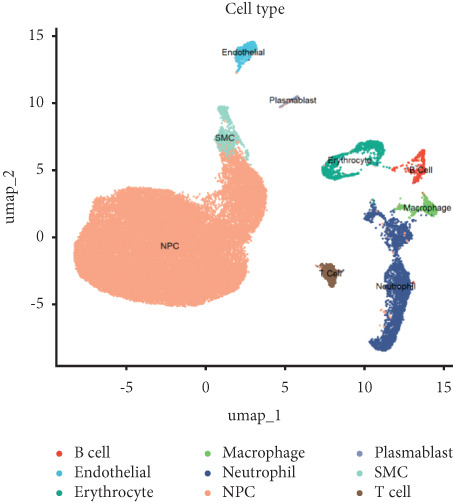
(b)

**Figure:**
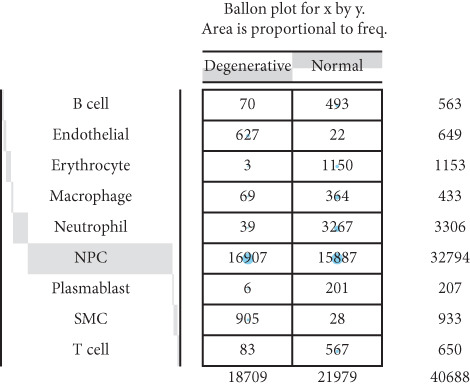
(c)

**Figure:**
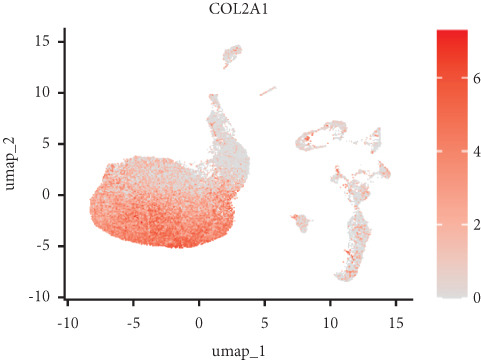
(d)

**Figure:**
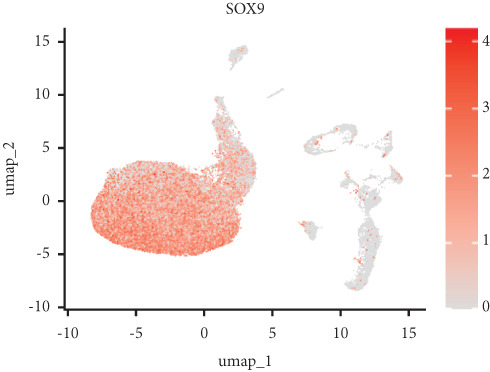
(e)

**Figure:**
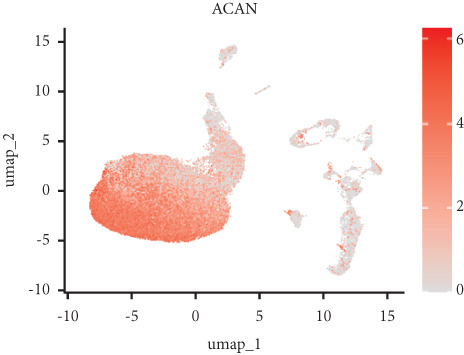
(f)

**Figure:**
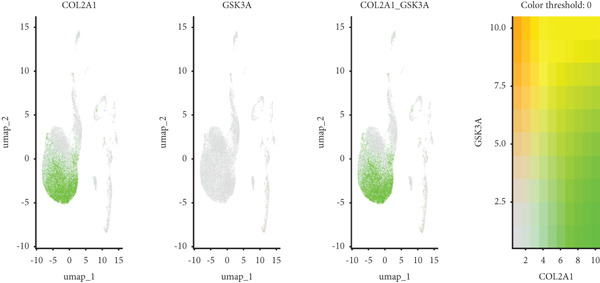
(g)

**Figure:**
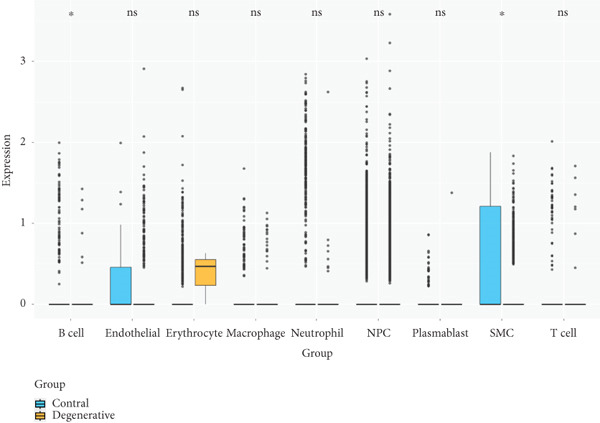
(h)

**Figure:**
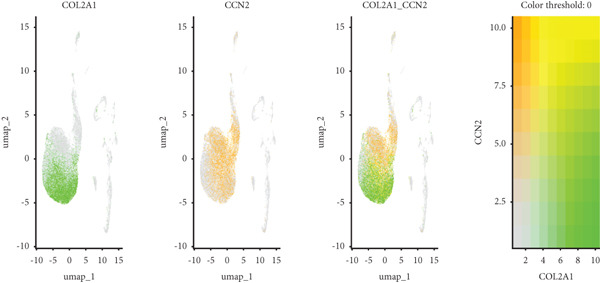
(i)

**Figure:**
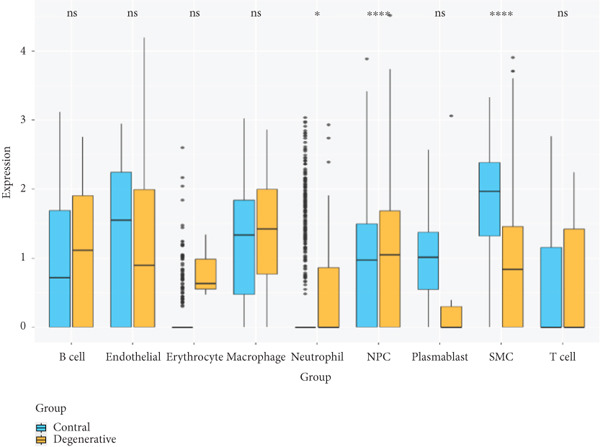
(j)

**Figure:**
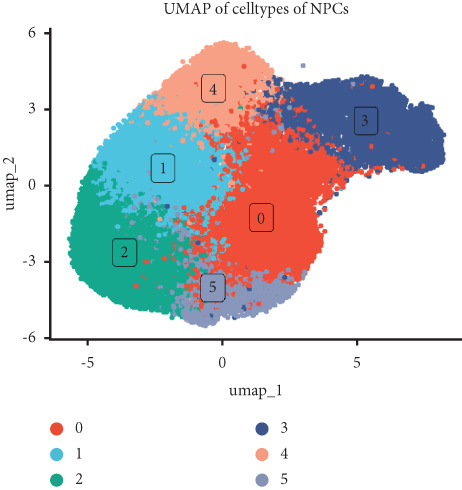
(k)

**Figure:**
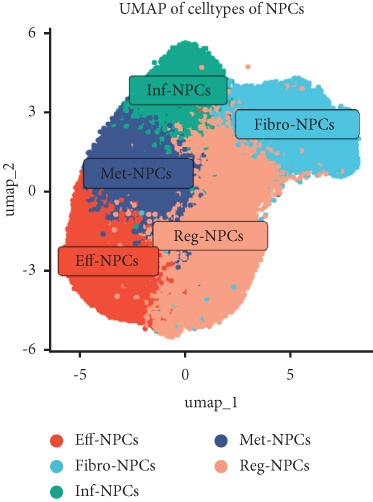
(l)

**Figure:**
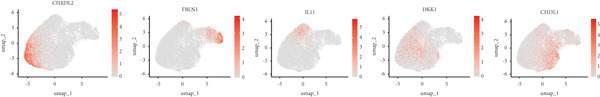
(m)

**Figure:**
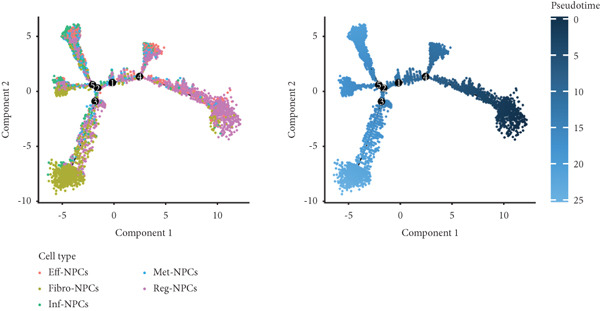
(n)

**Figure:**
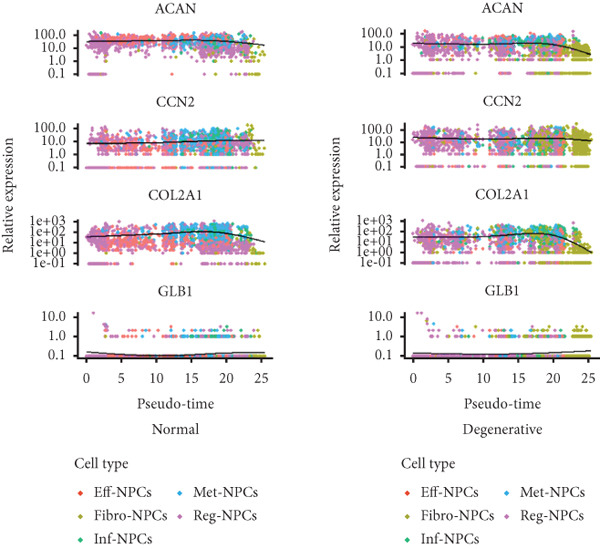
(o)

**Figure:**
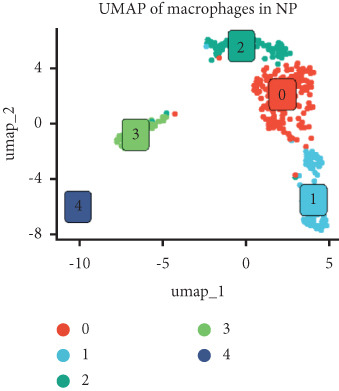
(p)

**Figure:**
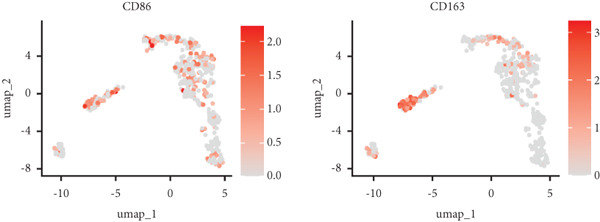
(q)

**Figure:**
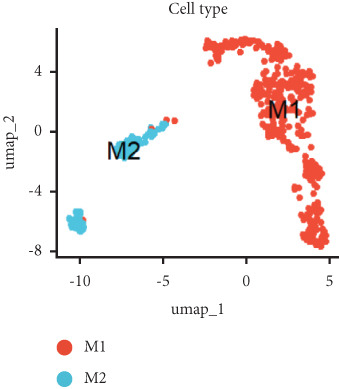
(r)

**Figure:**
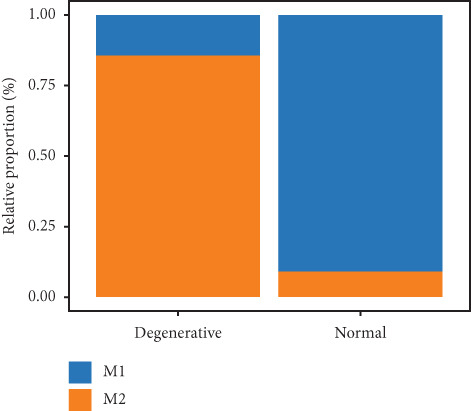
(s)

### 3.8. The Expression Levels of the Hub‐IVDD‐SRDEGs and Validation in Cell Models

From the gene expression matrix, it is evident that CTGF is upregulated in the degenerative group, while GSK3A is downregulated in the degenerative group (Figure 9a). We treated NPCs with IL‐1*β* to establish rat and human cell models of IVDD and used collagen II expression levels to verify the success of the models. It can be observed from the cell models that the protein expression levels of CTGF are consistent with the expression profiling by array results (Figure 9b). Observation under a 10× optical microscope revealed a significantly higher proportion of SA‐*β*‐gal‐positive blue‐stained cells in the IL‐1*β*‐induced degenerative group compared to controls, indicating enhanced cellular senescence characterized by elevated expression of the senescence marker SA‐*β*‐gal (Figure 9c).

Figure 9The expression levels of the two hub genes and verification in vitro. (a) The expression levels of CTGF and GSK3A in datasets.  ^∗∗∗^ *p* < 0.0001. (b) The CTGF expression levels in the human and rat cell models. (c) Senescence‐associated *β*‐galactosidase‐stained nucleus pulposus cells.

**Figure:**
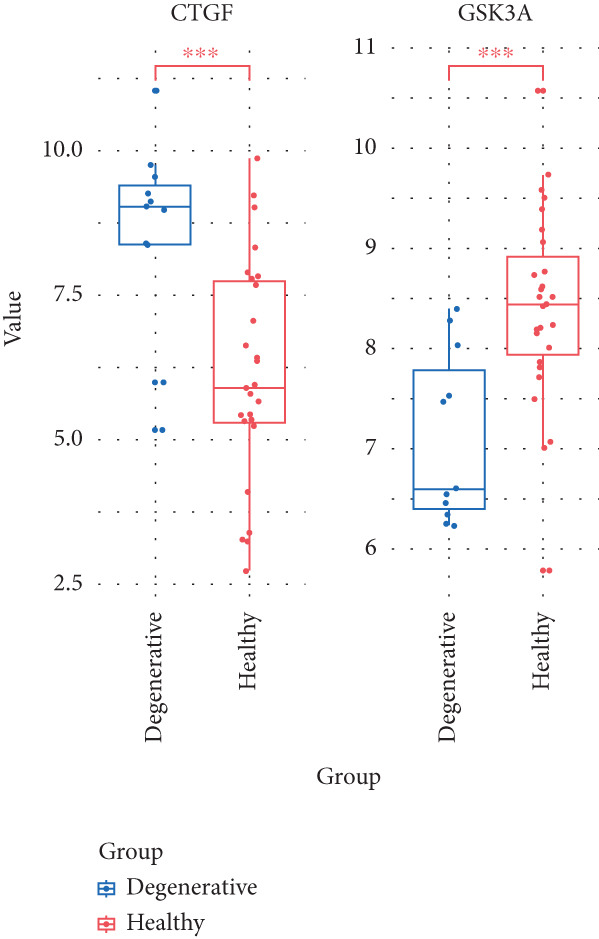
(a)

**Figure:**
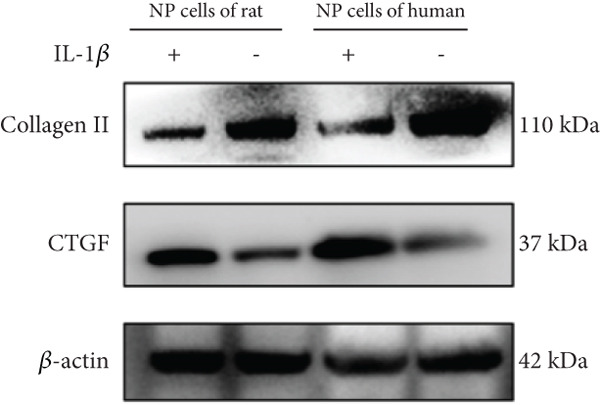
(b)

**Figure:**
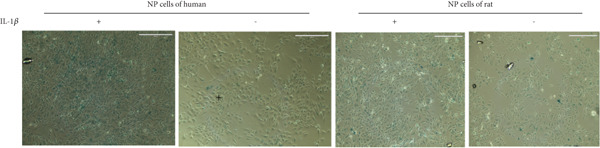
(c)

## 4. Discussion

With the extension of average life expectancy and working hours, it is expected that the number of patients with IVDD will increase annually [21]. The current preferred treatment for IVDD is conservative treatments (such as physical therapy and nonsteroidal anti‐inflammatory drugs), with surgery as the last resort [22]. Patients who undergo surgery are at risk of related complications. Stem cell therapy [23], tissue engineering [24], and gene therapy are still in their initial stages and are immature technologies. The pathogenesis of IVDD may involve inflammation [25, 26], mechanical load [27], and pyroptosis [28]. Therefore, there is an urgent need to explore and develop molecular and drug treatments that can alleviate symptoms and restore the stability of intervertebral disc tissue from a mechanistic perspective.

We downloaded expression profiling by array of intervertebral disc tissues from the GEO, merged them, and removed batch effects. The samples were categorized into control and degeneration groups according to the Thompson grade. DEG analysis was performed using the R package limma. The DEGs overlapped with 28 genes (SRDEGs) from the 543 SRGs obtained from the HAGR. WGCNA was conducted on the expression profiling, with the blue module showing the highest correlation with IVDD. Eight genes in the blue module intersected (IVDD‐SRDEGs) with 28 SRDEGs. Two machine learning algorithms (random forest and LASSO regression) were used to identify two hub‐IVDD‐SRDEGs (CTGF and GSK3A) from the eight IVDD‐SRDEGs. Nomogram and ROC curve were established to evaluate the diagnostic and predictive abilities of the two hub genes for IVDD. We analyzed scRNA‐seq data of NP tissues and observed that the expression of CTGF in NPCs was significantly higher in the degenerative group compared to the control group. This finding aligns closely with our results from bulk‐seq analyses. In contrast, the expression of GSK3A in NPCs was found to be extremely low, showing no significant differences between the control and degenerative groups. Furthermore, we validated the increased expression of CTGF in degenerative NPCs using a cellular model.

Senescence is an important nosogenesis for degenerative diseases. Despite the existence of various hypotheses and controversies regarding the mechanisms of cellular senescence, scholars generally acknowledge that DDR and the SASP are crucial tache in senescence [29]. Researchers like Le Maitre et al. have found an increased proportion of senescent cells in degenerated and aging intervertebral discs, with elevated expression levels of p16^INK4a^ (a cell cycle arrest protein positively correlated with senescence) [30]. Cell senescence has been confirmed to play a significant role in IVDD [31–33]. A study by Patil et al. has reported that increased expression of SASP such as IL‐6 and IL‐8 in degenerative intervertebral discs indicates the enhanced content of mitochondrial and ATP in senescent disc cells to meet the demands of SASP secretion and protein metabolism [31]. According to Che et al., the expression level of p16 is positively correlated with oxidative stress and DNA damage in NPCs, and the deficiency of p16 has a protective effect on IVDD in mice [34]. Patil et al. confirmed in mouse models that clearing p16^INK4a^ positive senescent cells can alleviate age‐related IVDD [35]. Research by Nasto et al. suggested that the NF‐*κ*B signaling pathway was closely associated with age‐related IVDD, and injecting NF‐*κ*B signaling pathway inhibitors into aging intervertebral discs of mice could increase the synthesis of proteoglycans in the intervertebral disc matrix [36].

CTGF/CCN2 is an effective inducer of tissue fibrosis and plays a crucial role in the processes of fibrosis in organs such as the lungs and liver [37]. CCN2 is the most studied member of the CCN family in intervertebral disc tissue, which is closely related to the generation of ECM [38, 39]. However, the precise mechanisms behind this function are not understood. CTGF plays an important role in the development and maturation of the notochord and notochord cells [40]. A study in transgenic mice has suggested that Smurf2 can accelerate IVDD in animal models by upregulating the expression of CTGF [41]. Mechanical stress has been reported to activate the RhoA/MRTF‐A signaling pathway, leading to the upregulation of CTGF expression and the deformation of NPCs [42]. Additionally, Sun et al. successfully differentiated fibrocartilage tissue (with high collagen I content) and hyaline‐like cartilage tissue (with high collagen II content) using 3D printing technology to deliver CTGF and TGF‐*β*3 mixed with bone marrow mesenchymal stem cells in the AF and NP regions of an IVDD scaffold [43]. Furthermore, an immunohistochemical study by Peng et al. on 43 degenerated intervertebral discs suggested that the expression of CTGF in degenerated discs from symptomatic patients was higher compared to asymptomatic individuals, while normal intervertebral discs showed no expression of CTGF [44]. These studies collectively indicate the significant role of CTGF in the development and degeneration of human intervertebral discs, with upregulated expression in degenerated discs, consistent with our research findings. We speculate that CTGF may promote the formation of collagen I and exhibit a negative correlation with the secretion of collagen II, leading to increased expression in degenerated NP tissue.

Recent research indicates that the inflammatory microenvironment plays a critical role in IVDD. Inflammatory cells and factors present in the degenerative intervertebral disc can initiate cascade reactions, leading to apoptosis of NPCs and degradation of the ECM [45]. Conversely, the infiltration of chronic inflammatory cells may also mediate the repair of damaged intervertebral disc tissue [46]. Some studies have reported that Pfirrmann Grades II and IV discs contain a similar number of M2 macrophages; however, Pfirrmann Grade II discs appear to have a higher proportion of intermediate macrophages. This suggests that slightly degenerated discs have an increased presence of M1 macrophages, which can subsequently differentiate into M2 macrophages, ultimately benefiting tissue repair [47]. Importantly, normal intervertebral disc tissue is characterized by poor vascularization. Nonetheless, our single‐cell analysis of the seven samples in GSE244889 revealed the presence of endothelial cells, SMCs, and erythrocytes, indicating that blood vessels infiltrate the NP tissue following degeneration. We identified infiltration of immune cells, including B cells, T cells, plasma cells, and macrophages, in both mildly and severely degenerated NP tissues, with a higher number of immune cells present in the mildly degenerative group compared to the severely degenerative group. These results suggest that an inflammatory reaction occurs following IVDD, with a substantial influx of inflammatory cells observed in the early stages. In contrast, as degeneration progresses, the degree of inflammatory cell infiltration diminishes alongside a reduction in the inflammatory response. Notably, some studies have indicated that levels of inflammatory cytokines, including IL‐6, IL‐1*β*, and IL‐17A, are significantly higher in younger patients compared to older individuals, suggesting that IVDD in younger patients may be linked to inflammation [17]. This correlates with our findings of heightened inflammatory cell infiltration in the context of mild IVDD. In severe IVDD, the observed reduction in immune cell infiltration may stem from either the activation of intrinsic tissue repair mechanisms or an immune‐exhausted state. Investigating dynamic alterations in immune cell populations during IVDD progression could elucidate the critical role of immunity in disease pathogenesis and inform novel strategies for diagnosis and therapeutic intervention.

Few studies prior to ours have combined different datasets of intervertebral discs and screened genes related to senescence. We integrated two datasets, including as many samples as possible, and used multiple methods to screen genes related to senescence. Finally, we verified the reliability of our results in scRNA‐seq and cell models. However, there are still some limitations:

1. The transcriptomic data was obtained from available public databases. Although we had integrated different datasets, it would be better to increase the sample size for higher credibility of results. Future studies should integrate multicenter cohorts to expand sample diversity and incorporate clinicopathological parameters.
2. We only validated the protein expression using scRNA‐seq and in vitro model. It would be preferable to validate the results in animal models and human discs in the future and to further investigate the mechanisms of gene action. Future work should employ conditional knockout animal models combined with ChIP‐seq and immunohistochemistry to delineate its downstream regulatory networks.
3. The identified biomarkers require diagnostic threshold optimization and prospective clinical validation to assess their sensitivity and specificity in independent cohorts. Furthermore, critical barriers remain in translating gene‐targeted therapies, including delivery systems (e.g., intradiscal injection of nanoparticle carriers), off‐target effects, and long‐term safety evaluations.

Future work will incorporate animal experiments and human tissue specimens to further dissect the senescence–immunity interplay.

## 5. Conclusion

We integrated two datasets (GSE23130 and GSE15227) and analyzed expression profiling by array from a total of 38 intervertebral disc samples. Through differential gene expression analysis, WGCNA, machine learning (LASSO regression and random forest algorithm), and so on, we identified two hub‐IVDD‐SRDEGs (CTGF and GSK3A) and conducted preliminary scRNA‐seq verification and experimental verification of our conclusions. CTGF has the potential for diagnosis and treatment of IVDD. In the early stages of IVDD, angiogenesis occurs, accompanied by the infiltration of immune cells such as T cells, B cells, plasma cells, and macrophages. However, as the degree of degeneration intensifies, the extent of immune cell infiltration diminishes, leading to a reduction in the inflammatory response. But further experimental research is needed to validate and elucidate their roles in IVDD. Future research will focus on elucidating the interplay between cellular senescence and immune cell infiltration, as well as investigating whether immune exhaustion plays a critical role in IVDD and repair processes.

## Disclosure

All authors have read and agreed to the published version of the manuscript.

## Conflicts of Interest

The authors declare no conflicts of interest.

## Author Contributions

Conceptualization: Q.X. and J.W. Methodology: Y.W. and D.L. Software: Q.X. Validation: Q.X., Z.L., A.W., and Y.G. Formal analysis: Q.X. and J.Q. Writing—original draft preparation: Q.X. and J.Q. Writing—review and editing: D.L. and J.W. Visualization: Q.X. Supervision: J.W. Project administration: J.W. Funding acquisition: J.W.

## Funding

This study was funded by the Wuxi Health and Family Planning Commission (10.13039/501100016308) (BJ2023015).

## Supporting information


**Supporting Information** Additional supporting information can be found online in the Supporting Information section. The original blots of Figure 9b.

## Data Availability

The datasets generated during and/or analyzed during the current study are available from the corresponding author upon reasonable request.

## References

[1] GBD 2017 Disease and Injury Incidence and Prevalence Collaborators , Global, Regional, and National Incidence, Prevalence, and Years Lived With Disability for 354 Diseases and Injuries for 195 Countries and Territories, 1990-2017: A Systematic Analysis for the Global Burden of Disease Study 2017, Lancet. (2018) 392, no. 10159, 1789–1858, 10.1016/s0140-6736(18)32279-7, 2-s2.0-85056201393, 30496104. 30496104 PMC6227754

[2] Zhao C. , Quan X. , He J. , Zhao R. , Zhang Y. , Li X. , Sun S. , Ma R. , and Zhang Q. , Identification of Significant Gene Biomarkers of Low Back Pain Caused by Changes in the Osmotic Pressure of Nucleus Pulposus Cells, Scientific Reports. (2020) 10, no. 1, 10.1038/s41598-020-60714-y, 32111963. PMC7048739 32111963

[3] Ekman M. , Jönhagen S. , Hunsche E. , and Jönsson L. , Burden of Illness of Chronic Low Back Pain in Sweden: A Cross-Sectional, Retrospective Study in Primary Care Setting, Spine. (2005) 30, no. 15, 1777–1785, 10.1097/01.brs.0000171911.99348.90, 2-s2.0-23244457916, 16094281. 16094281

[4] Balagué F. , Mannion A. F. , Pellisé F. , and Cedraschi C. , Non-Specific Low Back Pain , Lancet. (2012) 379, no. 9814, 482–491, 10.1016/s0140-6736(11)60610-7, 2-s2.0-84856639147. 21982256

[5] Pfirrmann C. W. , Metzdorf A. , Zanetti M. , Hodler J. , and Boos N. , Magnetic Resonance Classification of Lumbar Intervertebral Disc Degeneration, Spine. (2001) 26, no. 17, 1873–1878, 10.1097/00007632-200109010-00011, 2-s2.0-0035449038. 11568697

[6] Mok F. P. S. , Samartzis D. , Karppinen J. , Fong D. Y. T. , Luk K. D. K. , and Cheung K. M. C. , Modic Changes of the Lumbar Spine: Prevalence, Risk Factors, and Association With Disc Degeneration and Low Back Pain in a Large-Scale Population-Based Cohort, Spine Journal. (2016) 16, 32–41, 10.1016/j.spinee.2015.09.060, 2-s2.0-84951977889. 26456851

[7] Feng C. , Liu H. , Yang M. , Zhang Y. , Huang B. , and Zhou Y. , Disc Cell Senescence in Intervertebral Disc Degeneration: Causes and Molecular Pathways, Cell Cycle. (2016) 15, 1674–1684, 10.1080/15384101.2016.1152433, 2-s2.0-84978043780. 27192096 PMC4957599

[8] Bussian T. J. , Aziz A. , Meyer C. F. , Swenson B. L. , van Deursen J. M. , and Baker D. J. , Clearance of Senescent Glial Cells Prevents Tau-Dependent Pathology and Cognitive Decline, Nature. (2018) 562, 578–582, 10.1038/s41586-018-0543-y, 2-s2.0-85055427189. 30232451 PMC6206507

[9] Childs B. G. , Baker D. J. , Wijshake T. , Conover C. A. , Campisi J. , and van Deursen J. M. , Senescent Intimal Foam Cells Are Deleterious at All Stages of Atherosclerosis, Science. (2016) 354, 472–477, 10.1126/science.aaf6659, 2-s2.0-84992730363. 27789842 PMC5112585

[10] Jeon O. H. , David N. , Campisi J. , and Elisseeff J. H. , Senescent Cells and Osteoarthritis: A Painful Connection, Journal of Clinical Investigation. (2018) 128, 1229–1237, 10.1172/jci95147, 2-s2.0-85045033986. 29608139 PMC5873863

[11] Zhou J. , Huang J. , Li Z. , Song Q. , Yang Z. , Wang L. , and Meng Q. , Identification of Aging-Related Biomarkers and Immune Infiltration Characteristics in Osteoarthritis Based on Bioinformatics Analysis and Machine Learning, Frontiers in Immunology. (2023) 14, 1168780, 10.3389/fimmu.2023.1168780, 37503333. 37503333 PMC10368975

[12] Bodnar A. G. , Ouellette M. , Frolkis M. , Holt S. E. , Chiu C. P. , Morin G. B. , Harley C. B. , Shay J. W. , Lichtsteiner S. , and Wright W. E. , Extension of Life-Span by Introduction of Telomerase Into Normal Human Cells, Science. (1998) 279, no. 5349, 349–352, 10.1126/science.279.5349.349, 2-s2.0-0010045614, 9454332. 9454332

[13] Herranz N. and Gil J. , Mechanisms and Functions of Cellular Senescence, Journal of Clinical Investigation. (2018) 128, 1238–1246, 10.1172/jci95148, 2-s2.0-85045064986. 29608137 PMC5873888

[14] Coppé J.-P. , Desprez P.-Y. , Krtolica A. , and Campisi J. , The Senescence-Associated Secretory Phenotype: The Dark Side of Tumor Suppression, Annual Review of Pathology. (2010) 5, 99–118, 10.1146/annurev-pathol-121808-102144, 2-s2.0-77949881221. PMC4166495 20078217

[15] Birch J. and Gil J. , Senescence and the SASP: Many Therapeutic Avenues, Genes & Development. (2020) 34, 1565–1576, 10.1101/gad.343129.120. 33262144 PMC7706700

[16] Novais E. J. , Diekman B. O. , Shapiro I. M. , and Risbud M. V. , p16(Ink4a) Deletion in Cells of the Intervertebral Disc Affects Their Matrix Homeostasis and Senescence Associated Secretory Phenotype Without Altering Onset of Senescence, Matrix Biology. (2019) 82, 54–70, 10.1016/j.matbio.2019.02.004, 2-s2.0-85062804454. 30811968 PMC6708504

[17] Fan C. , Wang W. , Zilin Y. , Wang J. , Wei X. , Ji Z. , He W. , Hua D. , Wang W. , Yao L. , Deng Y. , Geng D. , Xiexing W. , and Mao H. , M1 Macrophage-Derived Exosomes Promote Intervertebral Disc Degeneration by Enhancing Nucleus Pulposus Cell Senescence Through LCN2/NF-*κ*B Signaling Axis, Journal of Nanobiotechnology. (2024) 22, 10.1186/s12951-024-02556-8. PMC11140985 38816771

[18] Gao Y. , Chen X. , Zheng G. , Lin M. , Zhou H. , and Zhang X. , Current Status and Development Direction of Immunomodulatory Therapy for Intervertebral Disk Degeneration, Frontiers in Medicine. (2023) 10, 1289642, 10.3389/fmed.2023.1289642. 38179277 PMC10764593

[19] Wang M. , Wang H. , Wang X. , Shen Y. , Zhou D. , and Jiang Y. , Identification of Cellular Senescence-Related Genes and Immune Cell Infiltration Characteristics in Intervertebral Disc Degeneration, Frontiers in Immunology. (2024) 15, 1439976, 10.3389/fimmu.2024.1439976. 39328407 PMC11424418

[20] He X. , Wei H. , Zhang Y. , Chen M. , Ding Y. , Yang H. , He F. , Qiaoli G. , and Shi Q. , Cellular Senescence in Skeletal Disease: Mechanisms and Treatment, Cellular & Molecular Biology Letters. (2023) 28, 10.1186/s11658-023-00501-5. PMC10612178 37891477

[21] Wang F. , Cai F. , Shi R. , Wang X.-H. , and Wu X.-T. , Aging and Age Related Stresses: A Senescence Mechanism of Intervertebral Disc Degeneration, Osteoarthritis and Cartilage. (2016) 24, 398–408, 10.1016/j.joca.2015.09.019, 2-s2.0-84958153660. 26455958

[22] Lin E.-Y. , Kuo Y.-K. , and Kang Y.-N. , Effects of Three Common Lumbar Interbody Fusion Procedures for Degenerative Disc Disease: A Network Meta-Analysis of Prospective Studies, International Journal of Surgery. (2018) 60, 224–230, 10.1016/j.ijsu.2018.11.009, 2-s2.0-85057275936. 30471365

[23] Sakai D. and Andersson G. B. J. , Stem Cell Therapy for Intervertebral Disc Regeneration: Obstacles and Solutions, Nature Reviews Rheumatology. (2015) 11, 243–256, 10.1038/nrrheum.2015.13, 2-s2.0-84926420130. 25708497

[24] Lv B. , Li L. , Liangcong H. , Cheng P. , Yiqiang H. , Xie X. , Dai G. , Mi B. , Liu X. , and Liu G. , Recent Advances in GelMA Hydrogel Transplantation for Musculoskeletal Disorders and Related Disease Treatment, Theranostics. (2023) 13, 2015–2039, 10.7150/thno.80615. 37064871 PMC10091878

[25] Chen F. , Jiang G. , Liu H. , Li Z. , Pei Y. , Wang H. , Pan H. , Cui H. , Long J. , Wang J. , and Zheng Z. , Melatonin Alleviates Intervertebral Disc Degeneration by Disrupting the IL-1*β*/NF-*κ*B-NLRP3 Inflammasome Positive Feedback Loop, Bone Research. (2020) 8, no. 1, 10.1038/s41413-020-0087-2, 32133213. PMC7028926 32133213

[26] Chen S. , Xiaohao W. , Lai Y. , Chen D. , Bai X. , Liu S. , Yongchao W. , Chen M. , Lai Y. , Cao H. , Shao Z. , and Xiao G. , Kindlin-2 Inhibits Nlrp3 Inflammasome Activation in Nucleus Pulposus to Maintain Homeostasis of the Intervertebral Disc, Bone Research. (2022) 10, no. 1, 5, 10.1038/s41413-021-00179-5, 35013104. 35013104 PMC8748798

[27] Jinna W. , Chen Y. , Liao Z. , Liu H. , Zhang S. , Zhong D. , Qiu X. , Chen T. , Deying S. , Ke X. , Wan Y. , Zhou T. , and Peiqiang S. , Self-Amplifying Loop of NF-*κ*B and Periostin Initiated by PIEZO1 Accelerates Mechano-Induced Senescence of Nucleus Pulposus Cells and Intervertebral Disc Degeneration, Molecular Therapy. (2022) 30, 3241–3256, 10.1016/j.ymthe.2022.05.021. 35619555 PMC9552911

[28] Zhang W. , Li G. , Luo R. , Jie Lei Y. , Song B. W. , Ma L. , Liao Z. , Ke W. , Liu H. , Hua W. , Zhao K. , Feng X. , Xinghuo W. , Zhang Y. , Wang K. , and Yang C. , Cytosolic Escape of Mitochondrial DNA Triggers cGAS-STING-NLRP3 Axis-Dependent Nucleus Pulposus Cell Pyroptosis, Experimental and Molecular Medicine. (2022) 54, 129–142, 10.1038/s12276-022-00729-9. 35145201 PMC8894389

[29] Basisty N. , Kale A. , Jeon O. H. , Kuehnemann C. , Payne T. , Rao C. , Holtz A. , Shah S. , Sharma V. , Ferrucci L. , Campisi J. , and Schilling B. , A Proteomic Atlas of Senescence-Associated Secretomes for Aging Biomarker Development, PLoS Biology. (2020) 18, no. 1, e3000599, 10.1371/journal.pbio.3000599, 31945054. 31945054 PMC6964821

[30] Le Maitre C. L. , Freemont A. J. , and Hoyland J. A. , Accelerated Cellular Senescence in Degenerate Intervertebral Discs: A Possible Role in the Pathogenesis of Intervertebral Disc Degeneration, Arthritis Research & Therapy. (2007) 9, R45, 10.1186/ar2198, 2-s2.0-34249788140. 17498290 PMC2206356

[31] Patil P. , Falabella M. , Saeed A. , Lee D. , Kaufman B. , Shiva S. , Croix C. S. , Van Houten B. , Niedernhofer L. J. , Robbins P. D. , Lee J. , Gwendolyn S. , and Vo N. V. , Oxidative Stress-Induced Senescence Markedly Increases Disc Cell Bioenergetics, Mechanisms of Ageing and Development. (2019) 180, 97–106, 10.1016/j.mad.2019.04.006, 2-s2.0-85064762740. 31002926

[32] Veroutis D. , Kouroumalis A. , Lagopati N. , Polyzou A. , Chamilos C. , Papadodima S. , Evangelou K. , Gorgoulis V. G. , and Kletsas D. , Evaluation of Senescent Cells in Intervertebral Discs by Lipofuscin Staining, Mechanisms of Ageing and Development. (2021) 199, 10.1016/j.mad.2021.111564, 111564, 34474077. 34474077

[33] Silwal P. , Nguyen-Thai A. M. , Mohammad H. A. , Wang Y. , Robbins P. D. , Lee J. Y. , and Vo N. V. , Cellular Senescence in Intervertebral Disc Aging and Degeneration: Molecular Mechanisms and Potential Therapeutic Opportunities, Biomolecules. (2023) 13, no. 4, 10.3390/biom13040686, 37189433. PMC10135543 37189433

[34] Che H. , Li J. , Li Y. , Ma C. , Liu H. , Qin J. , Dong J. , Zhang Z. , Xian C. J. , Miao D. , Wang L. , and Ren Y. , p16 Deficiency Attenuates Intervertebral Disc Degeneration by Adjusting Oxidative Stress and Nucleus Pulposus Cell Cycle, eLife. (2020) 9, 10.7554/eLife.52570, 32125276. PMC7065909 32125276

[35] Patil P. , Dong Q. , Wang D. , Chang J. , Wiley C. , Demaria M. , Lee J. , Kang J. , Niedernhofer L. J. , Robbins P. D. , Sowa G. , Campisi J. , Zhou D. , and Vo N. , Systemic Clearance of p16(INK4a) -Positive Senescent Cells Mitigates Age-Associated Intervertebral Disc Degeneration, Aging Cell. (2019) 18, e12927, 10.1111/acel.12927, 2-s2.0-85065879708. 30900385 PMC6516165

[36] Luigi A. , Nasto H.-Y. S. , Robinson A. R. , Tilstra J. S. , Clauson C. L. , Sowa G. A. , Ngo K. , Dong Q. , Pola E. , Lee J. Y. , Niedernhofer L. J. , Kang J. D. , Robbins P. D. , and Vo N. V. , ISSLS Prize Winner: Inhibition of NF-*κ*B Activity Ameliorates Age-Associated Disc Degeneration in a Mouse Model of Accelerated Aging, Spine. (2012) 37, no. 21, 1819–1825, 10.1097/BRS.0b013e31824ee8f7, 2-s2.0-84867020239, 22343279. 22343279 PMC3395770

[37] Tong Z. , Chen R. , Alt D. S. , Kemper S. , Perbal B. , and Brigstock D. R. , Susceptibility to Liver Fibrosis in Mice Expressing a Connective Tissue Growth Factor Transgene in Hepatocytes, Hepatology. (2009) 50, 939–947, 10.1002/hep.23102, 2-s2.0-70349235490. 19670427 PMC2737071

[38] Abbott R. D. , Purmessur D. , Monsey R. D. , Brigstock D. R. , Laudier D. M. , and Iatridis J. C. , Degenerative Grade Affects the Responses of Human Nucleus Pulposus Cells to Link-N, CTGF, and TGF*β*3, Journal of Spinal Disorders & Techniques. (2013) 26, E86–E94, 10.1097/BSD.0b013e31826e0ca4, 2-s2.0-84880046620. 22907063 PMC3548970

[39] Mark Erwin W. , Ashman K. , O′Donnel P. , and Inman R. D. , Nucleus Pulposus Notochord Cells Secrete Connective Tissue Growth Factor and Up-Regulate Proteoglycan Expression by Intervertebral Disc Chondrocytes, Arthritis and Rheumatism. (2006) 54, 3859–3867, 10.1002/art.22258, 2-s2.0-33845633749. 17136753

[40] Erwin W. M. , The Notochord, Notochordal Cell and CTGF/CCN-2: Ongoing Activity From Development Through Maturation, Journal of Cell Communication And Signaling. (2008) 2, 59–65, 10.1007/s12079-008-0031-5, 2-s2.0-61549094059. 19003520 PMC2648046

[41] Qiuqian W. and Huang J. H. , Ectopic Expression of Smurf2 and Acceleration of Age-Related Intervertebral Disc Degeneration in a Mouse Model, Journal of Neurosurgery. Spine. (2017) 27, 116–126, 10.3171/2016.11.Spine16901, 2-s2.0-85021773752. 28387615

[42] Song M. , Zhang Y. , Sun Y. , Kong M. , Han S. , Wang C. , Wang Y. , Derong X. , Qihao T. , Zhu K. , Sun C. , Li G. , Zhao H. , and Ma X. , Inhibition of RhoA/MRTF-A Signaling Alleviates Nucleus Pulposus Fibrosis Induced by Mechanical Stress Overload, Connective Tissue Research. (2022) 63, 53–68, 10.1080/03008207.2021.1952193. 34420462

[43] Sun B. , Meifei Lian Y. , Han X. M. , Jiang W. , Qiao Z. , and Dai K. , A 3D-Bioprinted Dual Growth Factor-Releasing Intervertebral Disc Scaffold Induces Nucleus Pulposus and Annulus Fibrosus Reconstruction, Bioactive Materials. (2021) 6, 179–190, 10.1016/j.bioactmat.2020.06.022. 32913927 PMC7451922

[44] Peng B. , Chen J. , Kuang Z. , Li D. , Pang X. , and Zhang X. , Expression and Role of Connective Tissue Growth Factor in Painful Disc Fibrosis and Degeneration, Spine. (2009) 34, E178–E182, 10.1097/BRS.0b013e3181908ab3, 2-s2.0-65549108935. 19247157

[45] Li Y. , Cao L. , Li J. , Sun Z. , Liu C. , Liang H. , Wang D. , and Tian J. , Influence of Microgravity-Induced Intervertebral Disc Degeneration of Rats on Expression Levels of p53/p16 and Proinflammatory Factors, Experimental and Therapeutic Medicine. (2019) 17, 1367–1373, 10.3892/etm.2018.7085. 30680015 PMC6327631

[46] Xiang H. , Zhao W. , Jiang K. , Jiangtao He L. , Chen W. C. , and Li Y. , Progress in Regulating Inflammatory Biomaterials for Intervertebral Disc Regeneration, Bioactive Materials. (2024) 33, 506–531, 10.1016/j.bioactmat.2023.11.021. 38162512 PMC10755503

[47] Zhao D.-W. , Cheng Q. , Geng H. , Liu J. , Zhang Y. , Cui J. , Liu C. , and Cheng L. , Decoding Macrophage Subtypes to Engineer Modulating Hydrogels for the Alleviation of Intervertebral Disk Degeneration, Advanced Science. (2024) 11, e2304480, 10.1002/advs.202304480. 37939288 PMC10767410

