# Supplementary material for: Identification of Core Senescence‐Related Genes and Characterization of Comprehensive Immune Landscape in Intervertebral Disc Degeneration
Source: Int J Genomics. 2025 Oct 22;2025:2521994. doi: 10.1155/ijog/2521994 (PMC12541284; doi:10.1155/ijog/2521994)
Supplement: Supplementary file 1 — Supporting Information Additional supporting information can be found online in the Supporting Information section. The original blots of Figure 9b. [file IJOG-2025-2521994-s001.pdf]

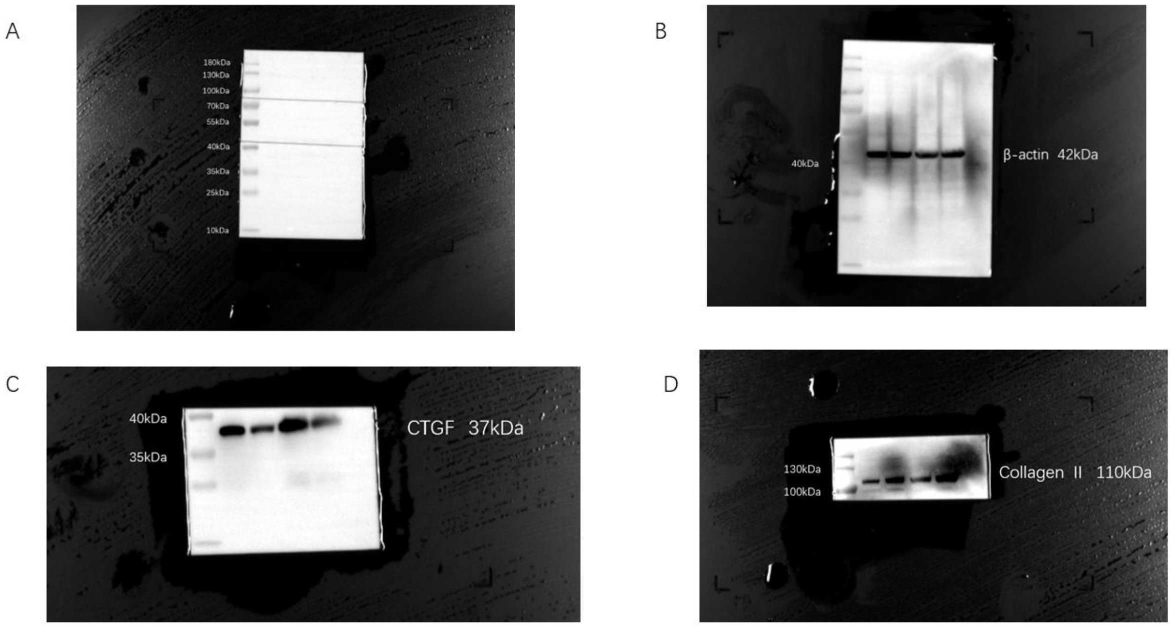

The original blots of Figure 9B. (A)Makes of WB. (B) Original blot of β-actin. (C) Original blot of CTGF. (D) Original blot of Collagen II.
